# A valid and reliable measure of nothing: disentangling the “Gavagai effect” in survey data

**DOI:** 10.7717/peerj.10209

**Published:** 2020-11-17

**Authors:** Victor B. Arias, Fernando P. Ponce, Martin Bruggeman, Noelia Flores, Cristina Jenaro

**Affiliations:** 1 Department of Personality, Assessment and Psychological Treatment, University of Salamanca, Salamanca, Spain; 2 School of Psychology, Pontificia Universidad Católica de Chile, Santiago, Chile

**Keywords:** Survey research, Validity, Validation, Measurement, Gavagai, Factor analysis

## Abstract

**Background:**

In three recent studies, Maul demonstrated that sets of nonsense items can acquire excellent psychometric properties. Our aim was to find out why responses to nonsense items acquire a well-defined structure and high internal consistency.

**Method:**

We designed two studies. In the first study, 610 participants responded to eight items where the central term (intelligence) was replaced by the term “gavagai”. In the second study, 548 participants responded to seven items whose content was totally invented. We asked the participants if they gave any meaning to “gavagai”, and conducted analyses aimed at uncovering the most suitable structure for modeling responses to meaningless items.

**Results:**

In the first study, 81.3% of the sample gave “gavagai” meaning, while 18.7% showed they had given it no interpretation. The factorial structures of the two groups were very different from each other. In the second study, the factorial model fitted almost perfectly. However, further analysis revealed that the structure of the data was not continuous but categorical with three unordered classes very similar to midpoint, disacquiescent, and random response styles.

**Discussion:**

Apparently good psychometric properties on meaningless scales may be due to (a) respondents actually giving an interpretation to the item and responding according to that interpretation, or (b) a false positive because the statistical fit of the factorial model is not sensitive to cases where the actual structure of the data does not come from a common factor. In conclusion, the problem is not in factor analysis, but in the ability of the researcher to elaborate substantive hypotheses about the structure of the data, to employ analytical procedures congruent with those hypotheses, and to understand that a good fit in factor analysis does not have a univocal interpretation and is not sufficient evidence of either validity nor good psychometric properties.

## Introduction

Imagine that we ask a person whether they agree with the following statement: *Aenean ut tortor imperdiet dolor scelerisque bibendum.* The phrase has no meaning (it is taken from Lorem Ipsum, which generates meaningless phrases that are often used to test visual effects in page layout). Perhaps the person gives a seemingly random answer, responds according to an arbitrary interpretation of the phrase, or even refuses to answer, thinking that we are joking. Suppose now that we build a set of ten items similar to this and compile the answers of 300 individuals. We will probably obtain a useless amalgam of meaningless data, with a weak structure and no internal consistency. Our “Lorem Ipsum scale” should not measure anything since arbitrary responses to incomprehensible questions can hardly reflect individual differences in trait or state.

However, Maul (2017) demonstrated in three studies that sets of items deliberately constructed to be uninterpretable, and in the “absence of a theory concerning what they measured and how they worked” (Maul, 2017, p.7), exhibited excellent psychometric properties (i.e., a robust factorial structure and high internal consistency). In the first study, which used a wording modification of Dweck’s growth mindset scale (2006) for 400 participants, the key noun in the original sentence (“intelligence”) was replaced by the nonsense word “gavagai” (e.g., “You can always substantially change how *gavagai* you are”; mindset-gavagai scale). The data showed a robust factorial structure, with 99% of the common variance explained by two correlated factors (*r* = 0.42) and high estimated reliability (alpha = 0.91). More importantly, the gavagai scale total scores correlated with Dweck’s growth mindset-intelligence scale (*r* = 0.41; *p* < 0.01) and certain personality traits (agreeableness: *r* = 0.09, *p* < 0.05; openness: *r* = 0.09; *p* < 0.05). As shown above, an item set composed of apparently meaningless items satisfied the usual requirements for the validation of psychological measures (internal consistency, robust dimensional structure, and correlation with other variables). However, it is possible to argue that responses are guided by the semantic context in which the term gavagai is inserted, allowing the participants to respond based on the idea that something is either malleable or fixed (Maul, 2017).

To address this, Maul designed two additional modifications (studies 2 and 3) with the aim of discarding potential semantic interpretations. The set of meaningless items was composed of statements worded using the Lorem Ipsum placeholder text (e.g., *Sale mollis qualisque eum id, molestie constituto ei ius*; Study 2) and items with no statements (e.g., “1.”; Study 3). Using the same analytic strategy, both studies revealed that these deliberately poorly designed item blocks resulted in a strongly unidimensional structure (92% and 98% of the common variance explained by the first factor for studies 2 and 3, respectively) and high reliability (alpha = 0.96 in both studies).

Based on these findings, Maul (2017) suggested that commonly used validation strategies failed to provide a falsifying test of the hypothesis that a given instrument measures a specific attribute, conferring a false appearance of validity and reliability. In fact, he mentioned that “favorable-looking results of covariance-based statistical procedures (…) should be regarded more as a default expectation for survey response data than as positive evidence for the validity of an instrument as a measure of a psychological attribute” (Maul, 2017, p.8). The results proposed by Maul are controversial, as they undermine the effectiveness of some of the standard procedures used to validate rating scales. In this respect, the study is percipient and provides arguments about the deficiencies and limitations of covariance-based statistical methods used to illustrate the validity of a measure. Additionally, Maul discussed the frequent lack of theoretical rigor in the explanations of the processes underlying the responses to items and the need to transcend fundamentally operationalist positions in the construction and interpretation of psychological measures.

While we agree with many of Maul’s claims, we also agree with concerns expressed by Rhemtulla, Borsboom & Van Bork (2017) that Maul’s results do not constitute sufficient evidence to conclude that traditional psychometric procedures, such as factor analysis, have severe usefulness problems. Based on their rationalization, there are two aspects that deserve to be mentioned.

First, traditional psychometric validation strategies have limitations. For example, Cronbach’s alpha has important limitations as an estimator of reliability and even internal consistency (e.g., Sijtsma, 2009). Similarly, regarding factor analysis, a good fit of the factor model does not guarantee that this procedure is the most appropriate strategy to explain the data structure (Rhemtulla, Van Bork & Borsboom, 2019), primarily when (a) no theoretical model supports a psychometric model that posits the existence of a continuous latent variable, and (b) the factor analysis was not designed to detect true nondimensional structures, so it is unlikely to falsify the hypothesis of the existence of a reflective latent variable.

The second aspect refers to participants’ highly structured responses to items intentionally designed to be uninterpretable. Rhemtulla, Borsboom & Van Bork (2017) suggested that apparently meaningless items would tap response processes. For instance, Maul’s response data can be explained by assuming that the participants replaced the word gavagai with a specific meaning. Additionally, these authors noted that an item set consisting of interchangeable versions of the same question would show high internal consistency and unidimensionality because “similar response processes result in similar responses to interchangeable questions” (p. 96; also referred to as “bloated specific” by Cattell, 1978). However, despite high internal consistency and unidimensionality, a set of interchangeable items may have questionable content validity and very limited usefulness.

### Response processes

At this point, the concept of response processes draws attention, and the question is whether the highly structured nature of the gavagai data is due to some unidentified response process. To hypothesize about the response processes underlying the phenomenon found by Maul, we can start from the cognitive model of the survey response process proposed by Tourangeau, Rips & Rasinski (2000). This model anticipates that an attentive respondent, after assigning a meaning to the question (comprehension stage), recalls pieces of information (e.g., facts, beliefs) from long-term memory (retrieval stage), accommodates the information in a decision (judgment stage), and decides an appropriate response (response stage). This model assumes both overlap between stages and backtracking from a later stage.

If the mindset-gavagai scale’s items were deliberately constructed to be uninterpretable, a gap is expected in the comprehension stage. This gap will prevent a consistent relationship between the meaning of the item and the meaning of the response, impeding the establishment of a causal connection between the item response and the respondent’s behavior. The puzzle is that although the key noun was unintelligible, Maul’s (2017) data showed a clear structure, similar to that observed in scales composed of understandable items, where such a relationship is expected.

One possible explanation is that there was a causal relationship between content and response, since the respondents attributed some meaning to gavagai, even if the meaning was personal and idiosyncratic. Moreover, the understandable terms in the items could have offered information that enabled the participants to assign a possible sense to the word gavagai (e.g., “... change how gavagai you are”, “... your gavagai level”). Finally, the eight items used by Maul were very similar to each other; they contained only one unintelligible term, and that term was always the same. Given these conditions, it is plausible that a respondent does not need much cognitive effort to assume a specific meaning of gavagai and to apply that meaning consistently in answering eight practically identical items. Under this assumption, similar responses to similar items will result in a robust variance–covariance matrix (with high internal consistency and clear factor structure), regardless of the meaning attributed to gavagai.

However, this notion does not apply to items composed of only gibberish words (such as those used by Maul in Study 2), since (a) gibberish words do not provide clues based on content (i.e., the items have no semantic polarity or understandable parts), and (b) the gibberish words differ across items and are organized as verbal units but with no sentence structure. In these circumstances, it is plausible to assume that the respondent will require considerable cognitive effort to assign meaning to each word (instead of one word for the whole scale) and then decide which answer is the most appropriate. Thus, in this low-information condition in which the participants are instructed to respond based on their gut instinct, it is unlikely that many people will dedicate the necessary effort to assigning meaning to each item and responding accordingly. Consequently, we should obtain response vectors closer to chance, resulting in a dataset with a weak internal structure. However, Maul’s reported findings in Study 2 seem to refute this hypothesis: even in a situation of low information, the data acquired a single-factor structure and high internal consistency.

To solve this problem, we may consider response processes in which the content of the items is irrelevant. Response style is a response process in which participants tend to respond consistently regardless of item content or directionality (e.g., owing to inattention, carelessness, low cognitive effort, or misunderstanding; Johnson, 2005; Meade & Craig, 2012; Curran, 2016). Two of the most common forms of content non-responsivity are straightlining (SL) and random responding (RR; see De Simone et al., 2018; Meade & Craig, 2012). SL is characterized by responses concentrated in a specific zone of the response scale, regardless of the item content and semantic polarity (i.e., acquiescence, disacquiescence, and midpoint responding). Once the reverse-keyed items are recoded, SL responses will produce distributions with little variance that are located in the center (midpoint response) or on one side of the scale (acquiescence/disacquiescence). In the RR style, the participant responds using the whole range of categories, giving rise to response vectors with high variability. However, non-content-based responses often acquire systematic patterns, even if people are instructed to respond randomly (e.g., Neuringer, 1986), so the term “pseudorandom” or “random-like” might be a more appropriate description of this response style. Both SL and RR patterns can generate significant but bogus statistical effects, spurious factors, and important alterations in the internal consistency and factor structure (Huang, Liu & Bowling, 2015; Maniaci & Rogge, 2014; Wood et al., 2017; Woods, 2006).

Based on this notion, it is possible to speculate that the highly structured data for gibberish items reported by Maul (2017) might hide a mixture of unordered latent categories. This possibility involves assuming that there is an alternative optimal structure of the data that the factor analysis is not able to detect (Rhemtulla, Van Bork & Borsboom, 2019). In the following paragraphs, we will develop this hypothesis by referring to relevant aspects of the data structure and assumptions about the relationship between the response to the item and the putative latent variable.

### Optimal data structure

Let us suppose that persons respond to gibberish items using response styles similar to the SL and RR strategies described above. The RR style generates response vectors distributed across a wide range of response categories. Conversely, the SL response style tends to generate distributions that are highly concentrated in one or two response categories (e.g., lower, medium, or upper), exhibiting low variance and high kurtosis. These two groups of respondents can be represented as independent classes, and a latent categorical model could be the best alternative to account for the entire dataset. The resulting classes may differ in their scale scores (e.g., disacquiescence will show lower scores). However, these scores do not reflect an individual-specific position on some continuous latent variable but a nominal distribution linked to membership within a particular class. The problem is that the joint distribution of all classes proffers a false impression of continuity.

To illustrate this possibility, we generated a dataset with four nominal classes (see Supplemental Material) that nevertheless have high internal consistency and perfectly fit a single-factor model. Figure S1 shows the mean response histograms of 10 items of five categories from 200 simulated response vectors organized into four unordered classes. These data are intended to roughly emulate the response styles described above. However, factor analysis conducted with both maximum likelihood robust (MLR) and weighted least square mean and variance adjusted (WLSMV) revealed high reliability (alpha = 0.87) and a great fit of a single-factor model regardless of the estimation method used (MLR [RMSEA <0.01; CFI = 1.000; TLI = 1.000]; WLSMV [RMSEA = 0.057; CFI = 0.970; TLI = 0.970]). These factor analysis results are evidently spurious, wrongly identifying a mixture of four classes as a single continuum.

Although factor analysis could not detect the categorical structure of simulated data, this does not mean that the method is flawed, because a mixture of classes can give rise to a correlation matrix that is compatible with what is expected from a factor. The problem is inherent not in factor analysis (which is one of several ways to model a covariance matrix) but in assuming that any dataset in a survey must necessarily have a dimensional structure. The solution is to take the data structure not as a predetermined assumption but as a research hypothesis (Markus, 2008a; Markus, 2008b; Markus & Borsboom, 2013). To examine this, we compared the fit of the factor model (continuous structure) with models that examine the presence of classes (categorical structure; cf., Lubke & Miller, 2015; Lubke & Neale, 2006; Lubke & Neale, 2008; De Boeck, Wilson & Acton, 2005). Table S1 shows the results of this analysis. Despite the excellent fit of the single-factor model presented above, fit indices recommended retaining the four-class categorical model, consistent with the four groups initially defined (98% of the cases were correctly classified). In the present study, we followed the same procedure to examine the optimal data structure.

### Relationship between the response to the item and the putative latent variable

If the response to an ordinal item involves a variation of an attribute, the association between them should be always in the same direction. This implies that, for example, responding “strongly agree” should mean a higher level of the attribute than responding “slightly agree,” and the empirical order of the response categories should be consistent with their theoretical order (after recoding the reverse-keyed items). If the response categories show a different empirical order, we could hypothesize that there is no latent continuous variable common to all respondents that governs their responses to the items.

Regarding response styles for responding to gibberish items, a constant relationship between these responses and the apparent underlying latent variable should not be expected. To test this, we contrasted the fit of a model that assumes a constant relationship (i.e., response categories always function as ordinal variables) with the fit of a model that does not (i.e., the empirical order of response categories may be different than expected).

### The present study

The purpose of the current study was to replicate the main results of the study by Maul (2017) and to explore the potential role of the response processes described above in the consistency and dimensionality of responses to meaningless items. To this end, we conducted two studies.

In the first study, we replicated and extended study 1 reported by Maul (2017) to evaluate the hypothesis that the highly structured dataset observed comes from responses guided by individual interpretations assigned to the word “gavagai.” We opted to ask participants to describe their interpretation of gavagai’s meaning (if any) and introduced this information into the analyses. We hypothesized that those participants who assigned a meaning to the key noun gavagai would consistently answer the mindset-gavagai item set and contribute to obtaining results similar to those previously reported by Maul (2017).

In the second study, we compared the structure and psychometric properties of three scales. The first scale was composed of intelligible words designed to assess extraversion (extraversion adjectives subscale from Goldberg, 1992 Big Five markers, e.g., extroverted, bold, talkative). The second scale comprised unintelligible and randomly generated nonexistent adjectives (e.g., cethonit, ormourse, wirington, which we henceforth call the “whatever” scale), randomly generated using a web application (https://randomwordgenerator.com/fake-word.php) in the absence of a specific theory. The third scale consisted of intelligible items but was designed to be uncorrelated and have neither dimensionality nor internal consistency (Greenleaf, 1992). The study aimed to explore the differences in the structure and properties of the data obtained from these instruments.

We hypothesized that both the extraversion and whatever scales would be highly structured, whereas the Greenleaf scale would not because it is composed of indicators with very disparate content (also suggested by Rhemtulla, Borsboom & Van Bork, 2017). Concerning the first two scales, we expected that the highly structured data would reflect the participants’ reflective responses (for the extraversion scale) or systematic response style (for the whatever scale). Based on this expectation, the two datasets should differ in the optimal data structure (continuous for extraversion and categorical for whatever) and the empirical versus the theoretical order of the response categories (consistent for extraversion and inconsistent for whatever).

## Study 1

### Materials & methods

#### Participants

The sample consisted of 610 participants (61% male) aged 18 to 75 years (Me = 34.7; Mdn = 32; SD = 11.7) who were recruited through the Prolific Academic (prolific.ac), an online crowdsourcing data tool that specializes in data collection for social and behavioral science research (cf. Palan & Schitter, 2018). All participants identified themselves as native English speakers and self-reported that they had been raised in the United States. They were compensated for their participation with $1.5 USD.

#### Measures

##### Dweck’s growth mindset scale (mindset-intelligence; Dweck, 2006).

This scale consisted of 8 balanced items about people’s beliefs about the extent to which intelligence is malleable (e.g., “You can change even your basic intelligence level considerably”) or fixed (e.g., “Your intelligence is something about you that you can’t change very much”), with a six-point response scale.

##### Maul’s growth mindset scale (mindset-gavagai; Maul, 2017).

This scale was identical to the mindset-intelligence scale except that the key noun (intelligence) was replaced with the term “gavagai” (e.g., “You can change even your basic gavagai level considerably”).

##### Open-ended question.

Participants responded to the questions “Did you assign any meaning to the word ‘gavagai’ while answering questions? If so, what was it?”

#### Data collection procedure

The protocol for data collection and analysis for this study was approved by the Institutional Review Board (IRB) of Pontificia Universidad Católica de Chile (ID: 180903005), and all procedures were performed in accordance with the ethical standards of the institutional and/or national research committees and the 1964 Helsinki Declaration and its later amendments or comparable ethical standards.

The responses were completely anonymous, and all participants gave express consent for their responses to be used in research. After agreeing to participate, each participant received the following instructions, which were identical to those used by Maul (2017, p.3):

“Thank you for taking this survey. You will be asked a number of questions regarding your opinions and viewpoints. We are interested in your intuitions—that is, your gut feelings—so please respond to each item based on your first reaction. There are no right or wrong answers. Even if you should encounter an unfamiliar word or phrase, please do not look it up—again, your gut reaction is what matters most.”

Next, the item blocks were presented in a predetermined order (gavagai items first) to control the potential effects of priming by the mindset-intelligence items. Each block was presented on a separate page, and within each block, the items were presented randomly to each participant to control for potential confirmatory bias (Weijters, Baumgartner & Schillewaert, 2013). Finally, the open question were presented.

#### Data analysis

To replicate the analyses performed by Maul (2017), we conducted exploratory factor analysis (EFA) using unweighted least squares (ULS) on Pearson’s correlation matrix. Then, we estimated Cronbach’s alpha and the correlation between the sum scores of the two scales. In addition, to examine the robustness of the EFA results, we examined the number of factors of both scales using parallel analysis (PA; Horn, 1965) and estimated the goodness of fit for these models using confirmatory factor analysis.

Next, we categorized the answers to the open-ended question about the meaning of gavagai as follows. Each response was read, and a preliminary set of codes was proposed according to the interpretation of the term “gavagai” (i.e., interpretations relating to personality, character, temperament, and analogous terms gave rise to a category called “personality”). Two judges independently reviewed all the responses and categorized them into the codes. Interjudge agreement was analyzed using Bangdiwala’s weighted agreement coefficient (B${}_{\mathrm{N}}^{\mathrm{W}}$; Bangdiwala, 1987), a weighted kappa statistic that takes into account different marginal frequencies in the data. B${}_{\mathrm{N}}^{\mathrm{W}}$ ranges from 0 (no agreement) to 1 (perfect agreement). All disagreements regarding the response classification were analyzed by a third reviewer and the two original reviewers to reach an agreement. After this process, those responses that were not assigned to any specific group (*n* = 18; 3.0%) were allocated to the category “miscellaneous.”

Finally, once the participants were assigned to the categories, we repeated the factor and reliability analyses described in the first step separately by category and compared the results with those obtained from the entire sample.

### Results

**Figure 1:**
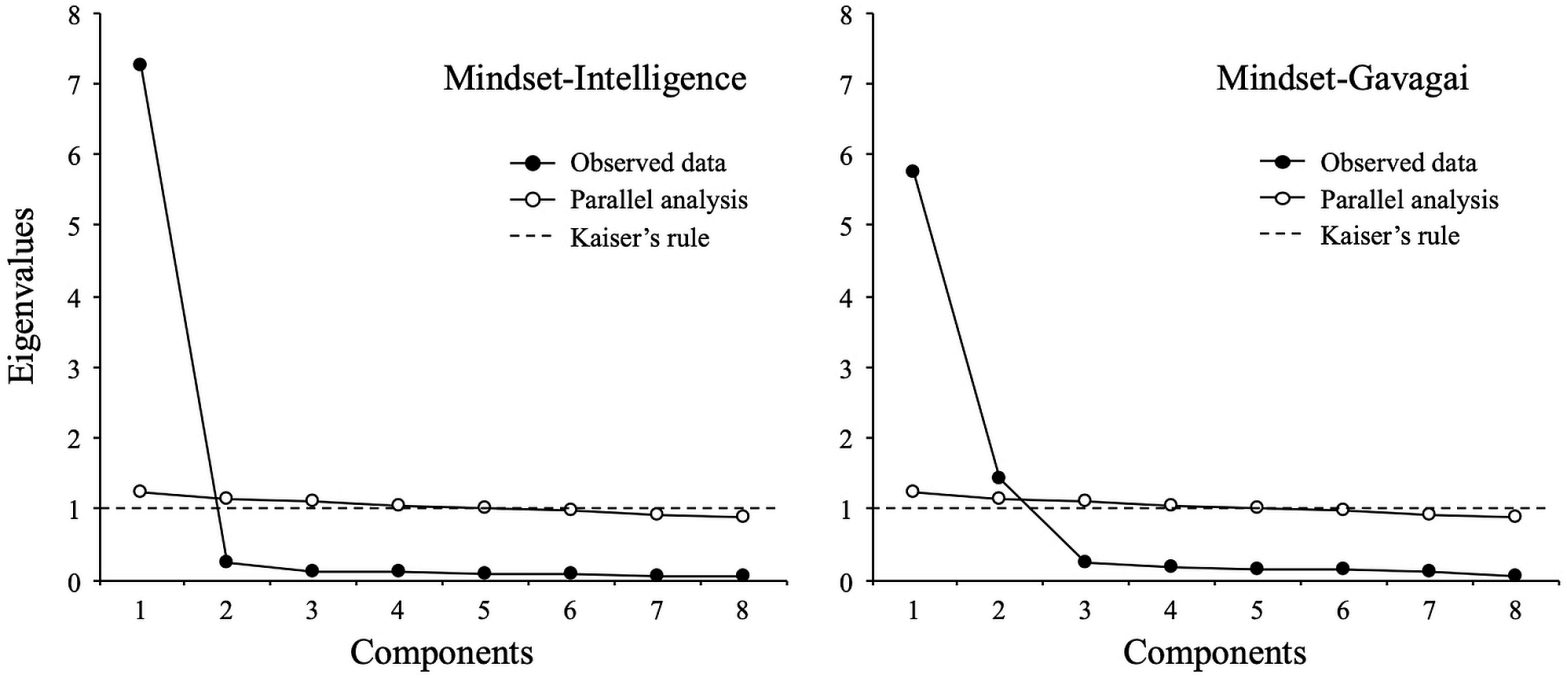
Parallel analysis (full sample).

Figure 1 depicts parallel analysis results by mindset condition (intelligence and gavagai). For the mindset-intelligence condition, PA and EFA suggested a single-factor solution that explained 85.3% of the common variance. The estimated eigenvalues of the first two factors were 7.26 and 0.25, suggesting that the first factor should be retained. For the mindset-gavagai condition, PA and EFA suggested a two-factor solution with eigenvalues of 5.74 and 1.43, compared to 0.23 for the third factor. Together, the two factors accounted for 83.3% of the common variance. The correlation between these two factors was *r* = 0.59.

**Table 1:** Factor loadings from exploratory factor analysis by mindsets condition.

**Item**	**Mindset items block**		
	Intelligence	Gavagai	
	**Factor 1**	**Factor 1**	**Factor 2**
You have a certain amount of *intelligence/gavagai*, and you can’t really do much to change it^a^	**0.922**	−0.006	**0.852**
Your *intelligence/gavagai* is something about you that you can’t change very much^a^	**0.926**	0.050	**0.855**
No matter who you are, you can significantly change your *intelligence/gavagai* level	**0.901**	**0.872**	0.003
To be honest, you can’t really change how *intelligent/gavagai* you are^a^	**0.930**	0.041	**0.846**
You can always substantially change how *intelligent/gavagai* you are	**0.924**	**0.898**	0.034
You can learn new things, but you can’t really change your basic *intelligence/gavagai*^a^	**0.887**	−0.036	**0.871**
No matter how much *intelligence/gavagai* you have, you can always change it quite a bit	**0.906**	**0.897**	0.011
You can change even your basic *intelligence/gavagai* level considerably	**0.902**	**0.907**	−0.009

**Notes.**

All loadings are standardized. Significant factor loadings (*p* < 0.05) appear in bold.

a Item is reverse-coded.

Table 1 shows the factor loadings of the EFA models. For the mindset-intelligence condition, the factor loadings were all above 0.85 (ranging between 0.877 and 0.930). For the mindset-gavagai condition, the structure was markedly two-dimensional, with positive and negative items grouped into distinct factors and with high primary loadings (between 0.846 and 0.907) and small cross-loadings (between 0.036 and 0.050).

Similar to the results reported by Maul (2017), the estimated reliabilities obtained in our study were quite high for the mindset-intelligence (alpha = 0.970) and the mindset-gavagai items blocks (alpha = 0.920). Finally, the correlation between the sum scores of the two item sets was 0.540 (*p* < 0.01).

According to these results, the mindset-gavagai scale exhibited a robust dimensional structure, explaining a substantial proportion of the common variance. Additionally, it showed a high internal consistency, and the sum score was moderately associated with the mindset-intelligence measure. Thus, we are able to conclude that our results approximate those reported previously by Maul (2017; Study 1).

With respect to the assessment of the statistical fit, we specified both a single-factor model for each scale (intelligence and gavagai) and a two-factor model for positively and negatively worded items. The overall fit statistics are reported in Table 2. For the mindset-intelligence scale, the results did not fully support the single-factor structure (CFI = 0.93; TLI = 0.90; RMSEA = 0.11), whereas the two-factor model showed an improved fit to the data (CFI = 0.99; TLI = 0.99; RMSEA = 0.03). However, in the two-factor model, the correlation between the factors was 0.94, a value high enough to suggest that the two dimensions are empirically indistinguishable. This inconsistency could be due to the presence of a limited number of highly incoherent response vectors; this phenomenon is commonly observed when using balanced scales (Podsakoff et al., 2003) such as those used here.

**Table 2:** Overall fit statistics for the growth mindset scales.

**Scale**	**Model**	**Chi-square (df)**	**RMSEA**	**CFI**	**TLI**	**SRMR**	**AIC**	**BIC**	**ECV**
Mindset-Intelligence	One-factor	189 (20)	0.118	0.934	0.907	0.023	10,749	10,855	
	Two-factor	29.5 (19)	0.030	0.996	0.994	0.008	10,424	10,534	
	RI-FA	28.4 (19)	0.029	0.996	0.995	0.009	10,422	10,532	0.97
Mindset-Gavagai	One-factor	653 (20)	0.228	0.608	0.451	0.148	12,482	12,588	
	Two-factor	16.5 (19)	0.000	1.000	1.000	0.014	11,312	11,423	
	RI-FA	14.8 (19)	0.000	1.000	1.000	0.013	11,309	11,419	0.80

**Notes.**

RMSEA Root Mean Square Error of Approximation CFI Comparative Fit Index TLI Tucker-Lewis Index SRMR Standardized root mean square residual AIC Akaike Information Criterion BIC Bayesian Information Criterion ECV Common variance explained by the trait factor in RI-FA models RI-FA Random intercept factor analysis

**Figure 2:**
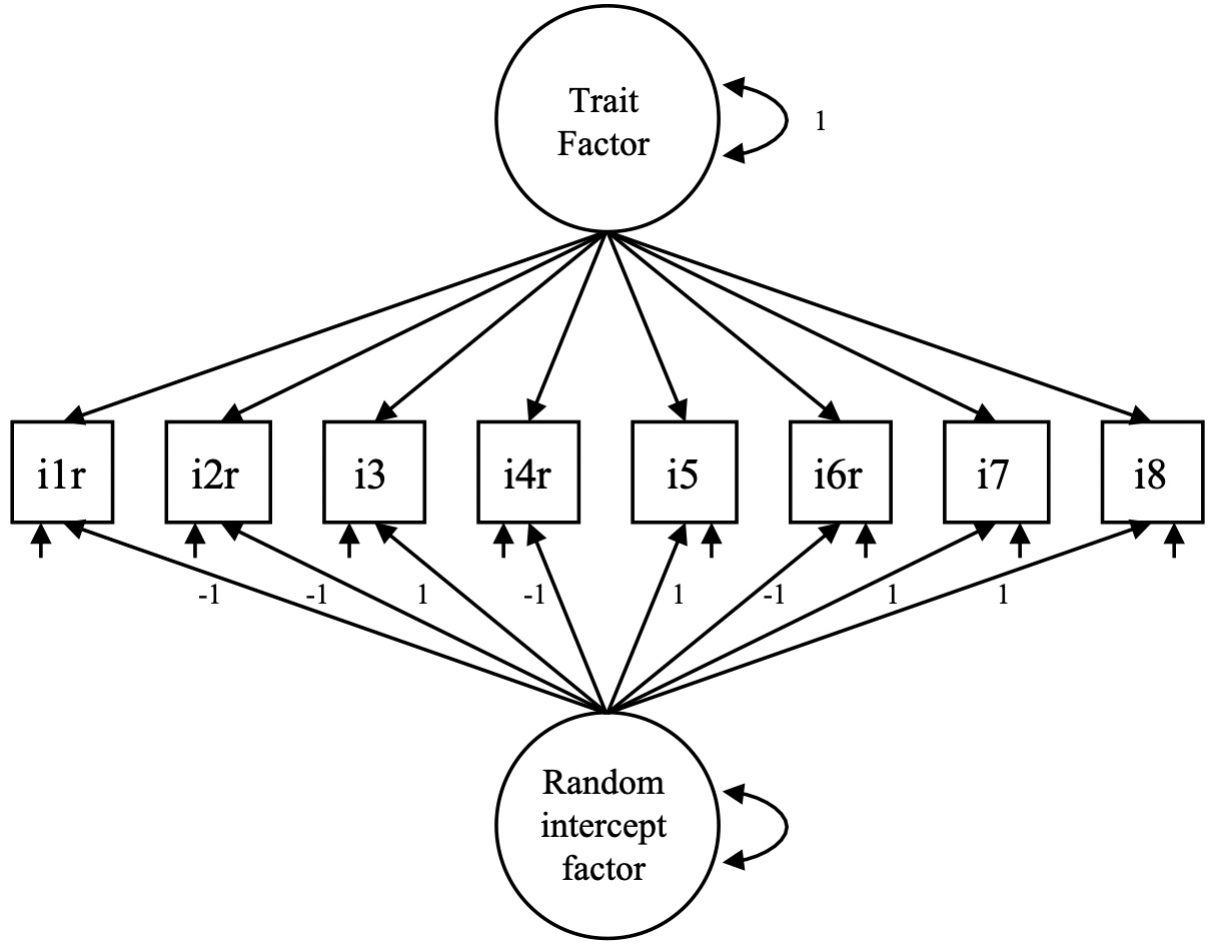
Specification of the random intercept factor model.

Inconsistent responses to positive and negative items can produce sets of spurious correlations that contribute to violating conditional independence and consequently reduce the goodness of fit for the single-factor model. To examine this, we also estimated a random intercept factor model (RI-FA; Maydeu-Olivares & Coffman, 2006). The RI-FA model consists of a trait factor plus a random intercept factor that captures the variance due to response inconsistencies in balanced scales. Figure 2 shows an example of a RI-FA model. All the trait factor loadings are freely estimated, and the factor variance is fixed at 1. The loadings of the RI-FA factor are fixed at 1 except for the reverse-keyed items, which are fixed at -1 (assuming that the responses have been recoded). The variance of the RI-FA factor is freely estimated. Finally, the correlation between factors is fixed at zero.

From the standardized factor loadings obtained with the RI-FA model, we estimated the explained common variance by the trait factor (ECV; Brunner, Nagy & Wilhelm, 2012). For the mindset-intelligence scale, the RI-FA model showed a marginally improved fit compared to the two-factor model (see Table 2). In addition, approximately 97% of the explained common variance was attributable to the trait factor (ECV = 0.97), whereas the random intercept factor captured only 3%. Thus, a significant proportion of the shared variance in the mindset-intelligence scale was caught by the trait factor, so we retained the unidimensional model as the most plausible structure.

For the mindset-gavagai scale, the single-factor model showed an unacceptable fit (CFI = 0.61; TLI = 0.45; RMSEA = 0.23), whereas the two-factor model showed an almost perfect fit (CFI = 1.00; TLI = 1.00; RMSEA <0.01) and a between-factor correlation of 0.61. However, the RI-FA model also showed good fit (CFI = 1.00; TLI = 1.00; RMSEA <0.01), with the trait factor explaining 80% of the common variance. In the absence of a theory concerning what the mindset-gavagai scale is intended to measure or the response processes involved, it is difficult to decide between a single-factor model corrupted by inconsistent responses and a genuine two-factor model.

Below, we summarize the results of the categorization of the answers to the open-ended question. With a good degree of agreement (*B*${}_{N}^{W}=0.944$), the categorization resulted in four groups. The first group (intelligence group; *n* = 77, 12.6%) was composed of participants who interpreted the key noun “gavagai” as “intelligence” (e.g., “intelligence,” “general intelligence,” “ability or intelligence”). The second group (personality group; *n* = 109, 17.9%) consisted of participants who interpreted gavagai as “personality” (e.g., “personality,” “inner personality,” “basic personality”). In the third group (“miscellaneous group”; *n* = 310; 50.8%), we grouped several answers that could not be assigned to a specific category with an appropriate sample size for separate analysis.^1^ 1The answers involved were considerably diverse, from particular features such as “self-esteem,” “grit,” or “spirituality” to subjects such as ”biology,” “physical appearance,” “the potential for change” to more bizarre interpretations such as “something cool,” “the rabitness” ([sic], we believe in a clear allusion to the origin of the term gavagai), “the mojo,” or “Gwyneth Paltrow.”Finally, the fourth group (null group; *n* = 114; 18.7%) contained participants who claimed not to have thought of any meaning or assigned any interpretation to the term gavagai (e.g., “nothing,” “not really,” “no,” “I responded all yes,” and “nothing but I tried to be consistent^2^ 2“consistent” is being treated in terms of responses (select a specific response category consistently) rather than consistency in the concept leading to the responses.”).

**Figure 3:**
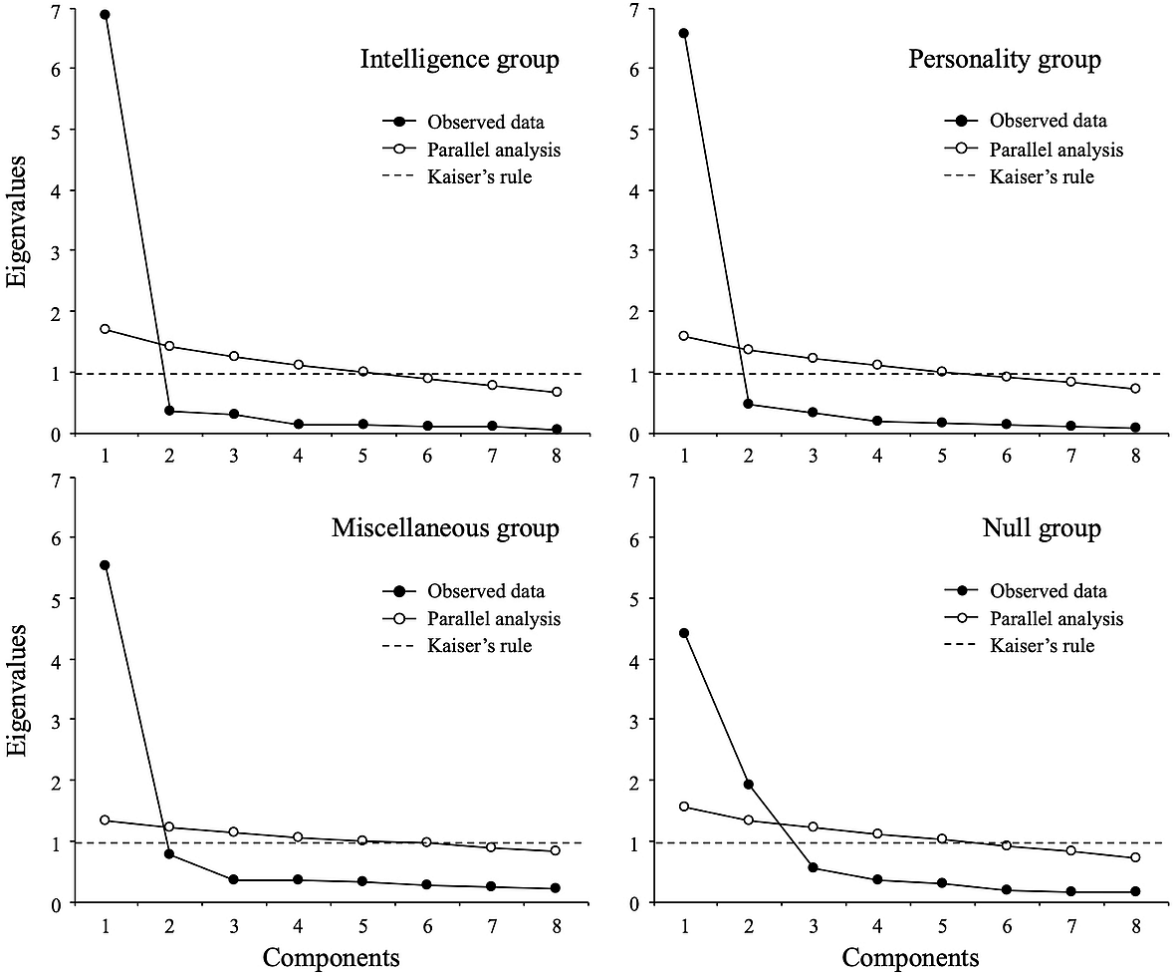
Parallel analysis (subsamples).

Figure 3 shows parallel analysis for the mindset-gavagai scale by the defined groups. For the groups intelligence, personality, and miscellaneous (i.e., those groups in which the participants reported assigning a specific meaning to gavagai; *n* = 496, 81.3%), the analysis suggested a unidimensional structure against the original two-dimensional solution obtained with the entire sample. For the null group, the analysis suggested a two-dimensional solution. Thus, the parallel analysis revealed that the initial two-factor structure found in the complete sample is consistent only with the solution obtained from those participants who were not able to assign a specific meaning to the word “gavagai”.

Table 3 shows the reliability coefficients for the mindset-gavagai scale and the correlation between the sum scores and the mindset-intelligence scale by group. For the intelligence and personality groups, Cronbach’s alpha was high and identical to the value reported using the mindset-intelligence scale (alpha = 0.97), and the association of these groups with the mindset-intelligence scale was also high (*r* = 0.94) or moderate (*r* = 0.65). These results are consistent with the notion that the participants assigned a meaning to the meaningless word gavagai. Indeed, the higher correlation in group intelligence may be due to the fact that the two scales are, according to those participants, the same scale.

**Table 3:** Cronbach’s alpha and correlations by group.

**Sample**	**Cronbach’salpha**	**Correlation with mindset- intelligence scores**
Intelligence group	0.97	0.94^**^
Personality group	0.97	0.65^**^
Miscellaneous group	0.94	0.43^**^
Null group	0.55	0.21^*^
Full sample	0.92	0.54^**^

**Notes.**

* *p* < 0.05.

** *p* < 0.01.

For the miscellaneous group, the reliability coefficient was also high (alpha = 0.94), and the correlation with the mindset-intelligence scale was moderately low (*r* = 0.43). This result is plausible because a pattern of consistent responses to the item block (within-respondents variability) about the malleability of different targets (between-respondents variability) could reduce the estimated magnitude of the general association with a fixed variable (intelligence). Finally, for the null group, Cronbach’s alpha was quite poor (alpha = 0.55) and showed a small association with the mindset-intelligence scale (*r* = 0.21). According to these results, the correlation in the full sample (*r* = 0.55) could be interpreted as the weighted average of correlations from a mixture of groups with different interpretations of the term “gavagai”.

Table 4 reports the overall model fit statistics for the three models previously evaluated by group. Since the factor extracted from the miscellaneous group probably represents an untraceable amalgam of interpretations of gavagai, for the sake of clarity, we presented only the results for the intelligence, personality, and null groups.^3^ 3The interested reader can obtain the analyses of the miscellaneous group from the first author or conduct the analyses using the raw data included as Supplemental Material.In all group conditions, the RI-FA model fitted better than others, although the proportion of common variance explained by both trait factor and random intercept factor differed across groups. For the intelligence and personality groups, the proportion of variance captured by the trait factor was high (95% and 94%, respectively), suggesting a negligible effect of the method variance. However, for the null group, a small proportion of the common variance was explained by the trait factor (31%), whereas the remaining 69% was attributable to the random intercept factor. Given that the random intercept factor in the RI-FA model is designed to capture spurious variance (Maydeu-Olivares & Coffman, 2006), the null group’s responses mostly constituted systematic noise without substantive interpretation.

**Table 4:** Fit statistics for the gavagai-mindset scales according to interpretation of the term “gavagai”.

**Group**	**Model**	Chi-square (df)	**CFI**	**TLI**	**RMSEA**	**SRMR**	**AIC**	**BIC**	**Corr**	**ECV**
Intelligence	One-factor	39 (20)	0.950	0.940	0.114	0.024	1,288	1,344		
	Two-factor	21 (19)	0.990	0.990	0.044	0.014	1,258	1,316	0.950	
	RI-FA	21 (19)	0.990	0.990	0.043	0.016	1,258	1,316		0.970
Personality	One-factor	51 (20)	0.950	0.920	0.120	0.028	1,654	1,719		
	Two-factor	26 (19)	0.980	0.980	0.060	0.018	1,615	1,682	0.940	
	RI-FA	25 (19)	0.990	0.980	0.056	0.019	1,614	1,681		0.970
Null	One-factor	90 (20)	0.750	0.650	0.176	0.180	2,564	2,629		
	Two-factor	12 (19)	1.000	1.000	0.000	0.057	2,400	2,469	−0.390	
	RI-FA	7 (19)	1.000	1.000	0.000	0.030	2.389	2,457		0.310

**Notes.**

CFI Comparative Fit Index TLI Tucker-Lewis Index RMSEA Root Mean Square Error of Approximation SRMR Standardized Root Mean Square of Residuals AIC Akaike Information Criterion BIC Bayesian Information Criterion Corr Inter-factor correlation in two-factor models ECV Common variance explained by the trait factor in RI-FA models RI-FA Random intercept factor analysis

Two main conclusions can be drawn from these results. First, the factor structure was substantially different between the group that claimed to have given an interpretation to the term “gavagai”, and the group that did not. In the first group the data acquired a strong unidimensional structure, while in the second group the data acquired a multidimensional structure, where most of the common variance was possibly due to non-substantive processes, such as respondents’ idiosyncratic usage of the response scale. Second, the correlations between the mindset-intelligence and mindset-gavagai scales varied substantially depending on the interpretation given to gavagai, from a near-perfect correlation in the group that interpreted gavagai as “intelligence”, to a very low correlation in the group that declared no interpretation. While further research is needed, this result suggests that different interpretations of gavagai led to different meaningful response processes.

## Study 2

### Material and methods

#### Participants

The sample consisted of 548 participants (51% male) aged 18 to 77 years (Me = 33.5; Mdn = 31; SD = 11.9) recruited through the Prolific Academic using the same procedures described in Study 1. All participants identified themselves as native English speakers and self-reported that they had been raised in the United States. They were compensated for their participation with $1.5 USD.

#### Measures

##### IPIP Big-Five factor markers (Goldberg, 1992).

We used the extraversion subscale consisting of 7 adjectives (i.e., extraverted, energetic, talkative, bold, active, assertive, and adventurous) to which the participants responded on a five-point accuracy scale (1 = Very inaccurate, 5 = Very accurate) according to the degree to which each adjective was applicable as a self-description.

##### Whatever scale.

This scale consisted of 7 fake “adjectives” (i.e., channed, leuter, cethonit, establechre, mystem, wirington, and ormourse), randomly generated using a web application (https://randomwordgenerator.com/fake-word.php), as a meaningless analog of Goldberg’s scale. The participants answered using the same five-point accuracy scale (1 = Very inaccurate, 5 = Very accurate).

##### Extreme response style measure (Greenleaf, 1992).

We included 7 items selected from the original scale,^4^ 4The items selected were “I am a homebody,” “Advertising insults my intelligence,” “Investing in the stock market is too risky for most families,” “I like to feel attractive to members of the opposite sex,” “My days seem to follow a definite routine—eating meals at the same time each day,” “A college education is very important for success in today’s world,” and “I will probably have more money to spend next year than I have now.”which were answered on a five-point scale (1 = Strongly disagree, 5 = Strongly agree) in accordance with the extent to which the participants agreed with each statement. From a broad set of indicators, Greenleaf selected those that minimized the correlation matrix (near zero). Therefore, this scale was not intended to measure anything but was originally designed to examine the extreme response style.

#### Data collection procedure

The protocol for data collection and analysis for this study was approved by the Institutional Review Board (IRB) of Pontificia Universidad Católica de Chile (ID: 180903005), and all procedures were performed in accordance with the ethical standards of the institutional and/or national research committees and the 1964 Helsinki Declaration and its later amendments or comparable ethical standards.

The responses were completely anonymous, and all participants gave express consent for their responses to be used in research. After agreeing to participate, each subject received an adapted version of Maul’s (2017) original instructions:

“Thank you for taking this survey. You will be asked a number of questions regarding your opinions about yourself. There are no right or wrong answers. Even if you should encounter an unfamiliar word, please do not look it up. We are interested in your intuitions—that is, your gut feelings—so please respond to unfamiliar words based on your first reaction.”

Next, the item blocks were presented in a balanced order. Half of the participants received the sequence whatever-Greenleaf-Goldberg, while the other half received the sequence Goldberg-Greenleaf-whatever. Each block was presented on a separate page, and within each block, the items were presented randomly to each participant to control for possible confirmatory bias (Weijters, Baumgartner & Schillewaert, 2013).

### Data analysis and results

Considering that the analysis consisted of a sequence of three concatenated phases and to facilitate reading, both the analytic procedure and the results are presented by phase.

#### Phase 1: Replication of Maul’s results

**Figure 4:**
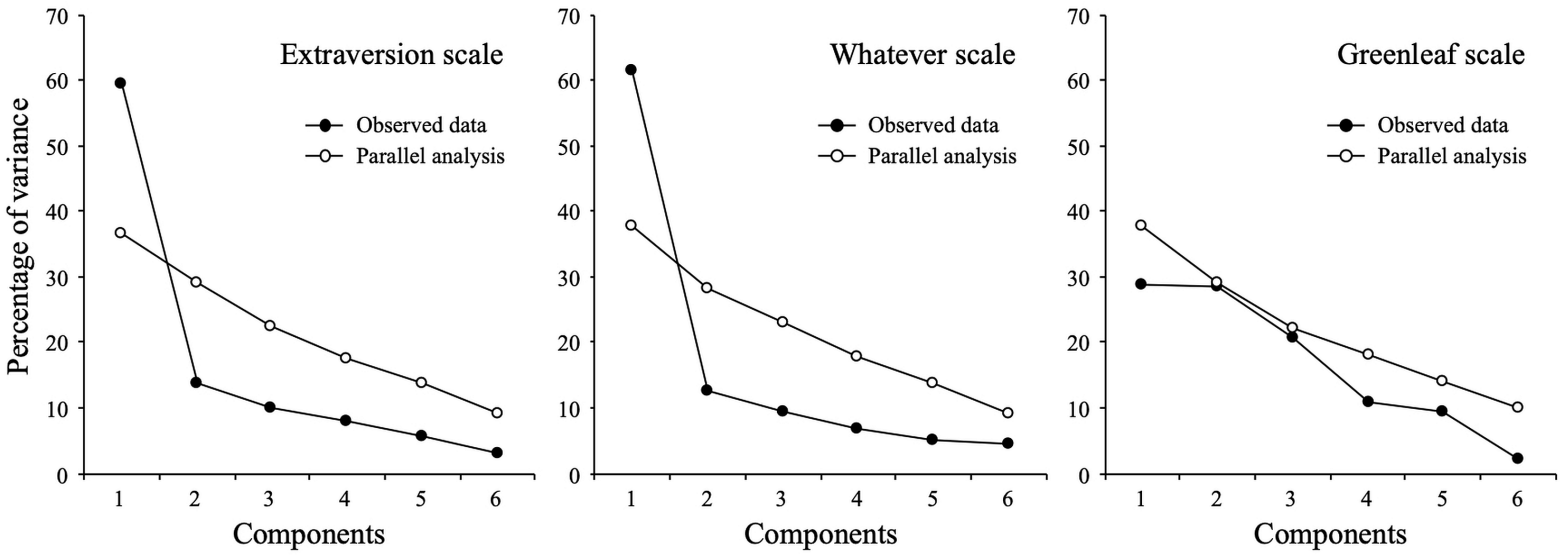
Parallel analysis.

First, we examined the data by estimating dimensionality through exploratory factor analysis, parallel analysis and internal consistency through Cronbach’s alpha (analytic procedures applied by Maul (2017) and our Study 1). Figure 4 shows the optimized parallel analysis by scale (Timmerman & Lorenzo-Seva, 2011). For the extraversion items, the analysis recommended retaining a single factor. Exploratory factor analysis (using ULS as the estimation method) resulted in a single-factor solution with eigenvalues for the first and second components of 3.67 and 0.94, respectively. This factor explained 52.4% of the common variance, and the standardized loadings ranged between 0.59 and 0.76. Cronbach’s alpha was 0.91. For the whatever scale, both parallel analysis and EFA also suggested a single-factor solution (eigenvalues for the first two components were 3.05 and 0.80), explaining 43.4% of the common variance, and factor loadings ranged between 0.53 and 0.65. Cronbach’s alpha was 0.89. Finally, for the Greenleaf scale, simulated eigenvalues from parallel analysis outperformed empirical values; therefore, there is no evidence of any factor structure.

#### Phase 2: Estimation of confirmatory models

Table 5 shows the confirmatory factor analysis results (robust maximum likelihood). For the extraversion scale, the results did not support the single-factor model (CFI = 0.85; TLI = 0.78; RMSEA = 0.15), suggesting either problems with the scale properties or misspecification of the model. Extraversion has generally been conceived as a major dimension that contains several facets (Johnson, 2014). Goldberg (1992) selected these items to maximize content validity. Therefore, it is reasonable to hypothesize a set of residual correlations between items from the same facet beyond the general factor of extraversion.

**Table 5:** Results from confirmatory factor analysis.

**Scale**	**Model**	**Chi-square**	**df(FP)**	**CFI**	**TLI**	**RMSEA**	**SRMR**
Extraversion	One-factor	174	14(21)	0.850	0.780	0.145	0.059
	One-factor (CUs)	54	10(25)	0.960	0.910	0.090	0.031
Whatever	One-factor	16	14(21)	0.990	0.990	0.019	0.026

**Notes.**

CUs Correlated uniqueness df(FP) Degrees of freedom (free parameters) CFI Comparative Fit Index TLI Tucker-Lewis Index RMSEA Root Mean Square Error of Approximation SRMR Standardized Root Mean Square of Residuals

With this hypothesis in mind, we examined both the modification indices (MI) and standardized expected parameter change for model modification (SEPC; Saris, Satorra & VanderVeld, 2009; Whittaker, 2012) to identify local sources of misfit. Four pairs of items showed correlated residuals with MI >10 and SEPC >0.20 (extraverted/talkative, assertive/bold, and energetic/active/adventurous). Considering that these correlations might reflect narrow facets of extraversion (probably warmth, assertiveness, and activity), we thought respecifying the model by releasing this set of correlations was justifiable. The fit improved considerably (CFI = 0.96; TLI = 0.91; RMSEA = 0.09), although this result, especially in RMSEA, remains far from satisfactory. The reasons for the discrepancy between RMSEA and CFI should be investigated (Lai & Green, 2016), but for reasons of brevity, we will consider this analysis sufficient and continue with the next. For the whatever scale, the fit results supported the unidimensional structure (CFI = 0.99; TLI = 0.99; RMSEA = 0.019), and no MI values were higher than 10, thus discarding the presence of any relevant local misfit.

#### Phase 3: Reinspection of the data and comparison with alternative structures

At this point, we had replicated the findings reported by Maul (2017) and found evidence of (a) an extraversion scale reflecting a theoretically continuous latent variable, with an acceptable fit only after introducing post hoc modifications, and (b) a meaningless item set that, surprisingly, seems to measure some kind of latent continuum (whateverness?) very well. Based on the hypothesis described above, our final set of analyses aimed to compare the statistical fit assuming the latent variable as a continuous latent trait (factor analysis) and as a categorical grouping variable (latent class analysis [LCA]; Hagenaars & McCutcheon, 2002; Mislevy & Verhelst, 1990).

The results of the factor analysis and latent class analysis are shown in Table 6. For the extraversion scale, the single-factor model was superior to the latent class models, whereas the 3-class model was, according to BIC and VLMR, the best latent class solution. For the whatever scale, the best fit was obtained with the latent class models, surpassing the unidimensional model in all cases. BIC and VLMR supported the three-class model.

**Table 6:** Results for internal structure analysis by scale.

**Scale**	**Model**	**FP**	**AIC**	**BIC**	**Entropy**	**VLMR**
Extraversion	**CFA One-factor**	**35**	**9,912**	**10,063**		
	LCA 2-Classes	57	10,349	10,594	0.81	<0.001
	LCA 3-Classes	86	10,093	10,464	0.79	<0.001
	LCA 4-Classes	115	9,985	10,480	0.80	0.760
	LCA 5-Classes	144	9,912	10,532	0.82	0.770
	LCA 6-Classes	173	9,869	10,614	0.83	0.760
Whatever	CFA One-factor	35	8,598	8,749		
	LCA 2-Classes	57	7,883	8,129	0.93	<0.001
	**LCA 3-Classes**	**86**	**7,330**	**7,701**	**0.95**	**<0.001**
	LCA 4-Classes	115	7,271	7,766	0.86	0.790
	LCA 5-Classes	144	7,247	7,867	0.87	0.770
	LCA 6-Classes	173	7,237	7,982	0.87	0.760

**Notes.**

FP Free parameters AIC Akaike Information Criterion BIC Bayesian Information Criterion VLMR Vuong-Lo-Mendell-Rubin likelihood ratio test

Retained model appear in bold.

**Figure 5:**
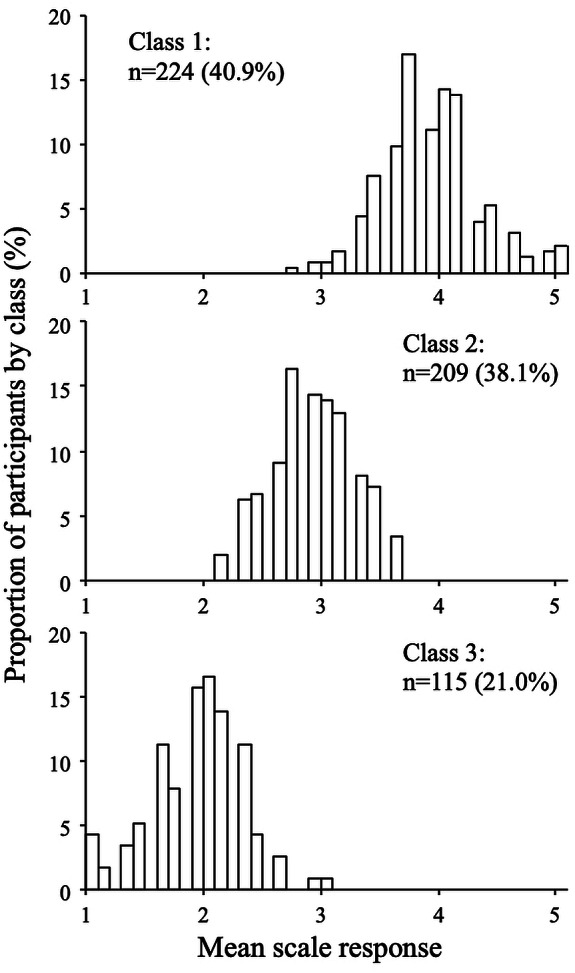
Response distributions by class (extraversion scale, three-class model).

**Figure 6:**
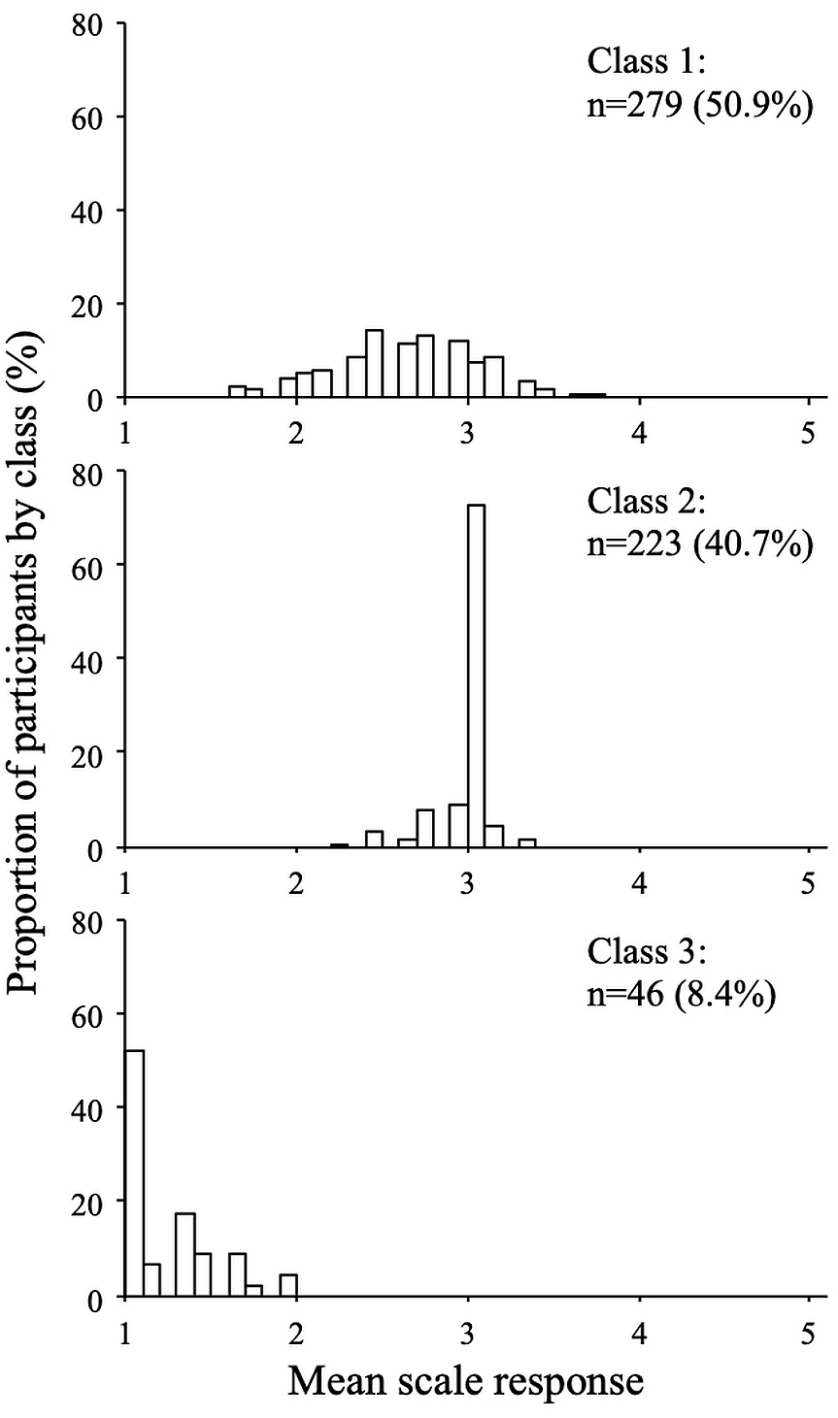
Response distributions by class (wathever scale, three-class model).

The distributions of the mean scale response for each class are shown in Figs. 5 and 6 (for the extraversion and whatever scales, respectively). The extraversion classes showed an ordered set of distributions that can be interpreted as a high extraversion class (*n* = 224; 40.9%), a moderate extraversion class (*n* = 209; 38.1%), and a low extraversion class (*n* = 115; 21.0%). This result is expected from fitting continuous multivariate data to a categorical model. For the whatever scale, the first class (*n* = 279; 50.9%) showed a dispersed distribution among the mean scale scores, whereas the second (*n* = 223; 40.7%) and the third class (*n* = 46; 8.4%) showed distributions concentrated around categories 1 and 3, respectively. The response distributions for these classes are quite similar to those expected from the random, straightline-disacquiescent, and straightline-midpoint response styles. We compared the response frequencies observed in the random response (RR) class with those calculated from a set of simulated uniform random data. The frequencies of each response category in the RR class (187, 867, 501, 363, and 35 from category 1 to category 5) were very different from those obtained from the random data (377, 383, 422, 390, and 381), suggesting that the RR class did not respond in a truly random way.

Finally, to examine the relation between the underlying trait (i.e., extraversion and whateverness) and the response categories, we compared the fit of a graded response model (GRM) that assumes that the response categories are ordinal (Samejima, 1969) and a nominal model (Bock, 1997) that allows unordered categories to be estimated even if the response categories are initially defined as ordinal. The fit for the graded and nominal IRT models is shown in Table 7.

**Table 7:** Results from IRT analysis.

**Scale**	**Model**	**−2LL**	**AIC**	**BIC**	**M**_**2**_	**RMSEA**
Extraversion	**Graded**	**9,842**	**9,912**	**10,063**	**832.5**	**0.05**
	Nominal	9,895	10,007	10,248	1,003.3	0.06
Whatever	Graded	8,528	8,598	8,749	2,914.0	0.12
	**Nominal**	**7,291**	**7,403**	**7,645**	**758.1**	**0.05**

**Notes.**

−2LL −2 LogLikelihood AIC Akaike Information Criterion BIC Bayesian Information Criterion M_**2**_ Limited Information Test Statistic (Maydeu-Olivares & Joe, 2005; Maydeu-Olivares & Joe, 2006) RMSEA Root Mean Square Error of Approximation

Retained model appear in bold.

**Figure 7:**
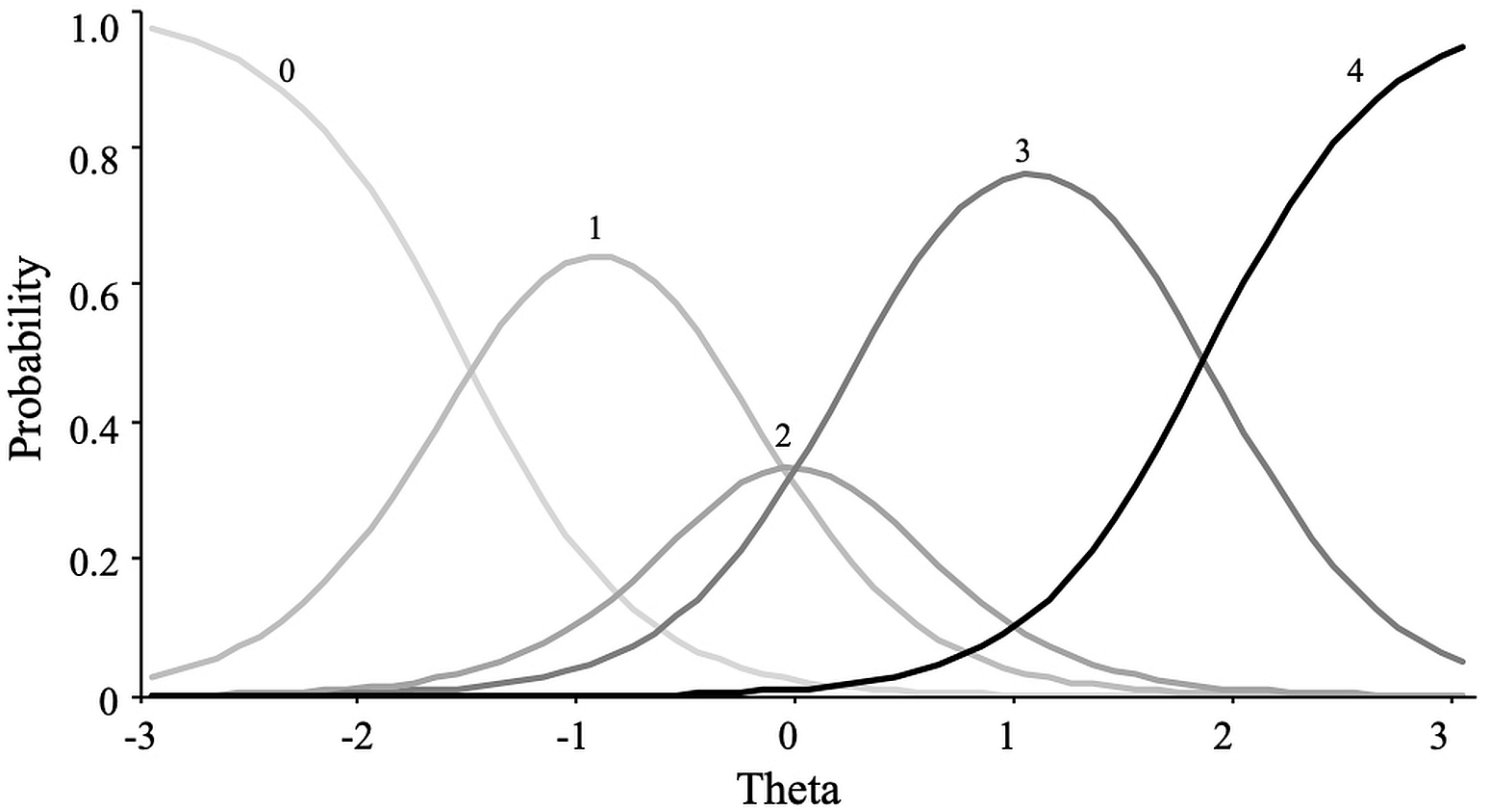
Item category curves of item “Bold”.

For the extraversion scale, the graded response model showed the best fit. This result implies that constraining the response categories to a constant relationship with the latent variable did not produce misfit, rejecting the hypothesis of an absence of dominance relationship. Figure 7 shows how response category probability curves for the item “bold” were empirically ordered according to response categories.

**Figure 8:**
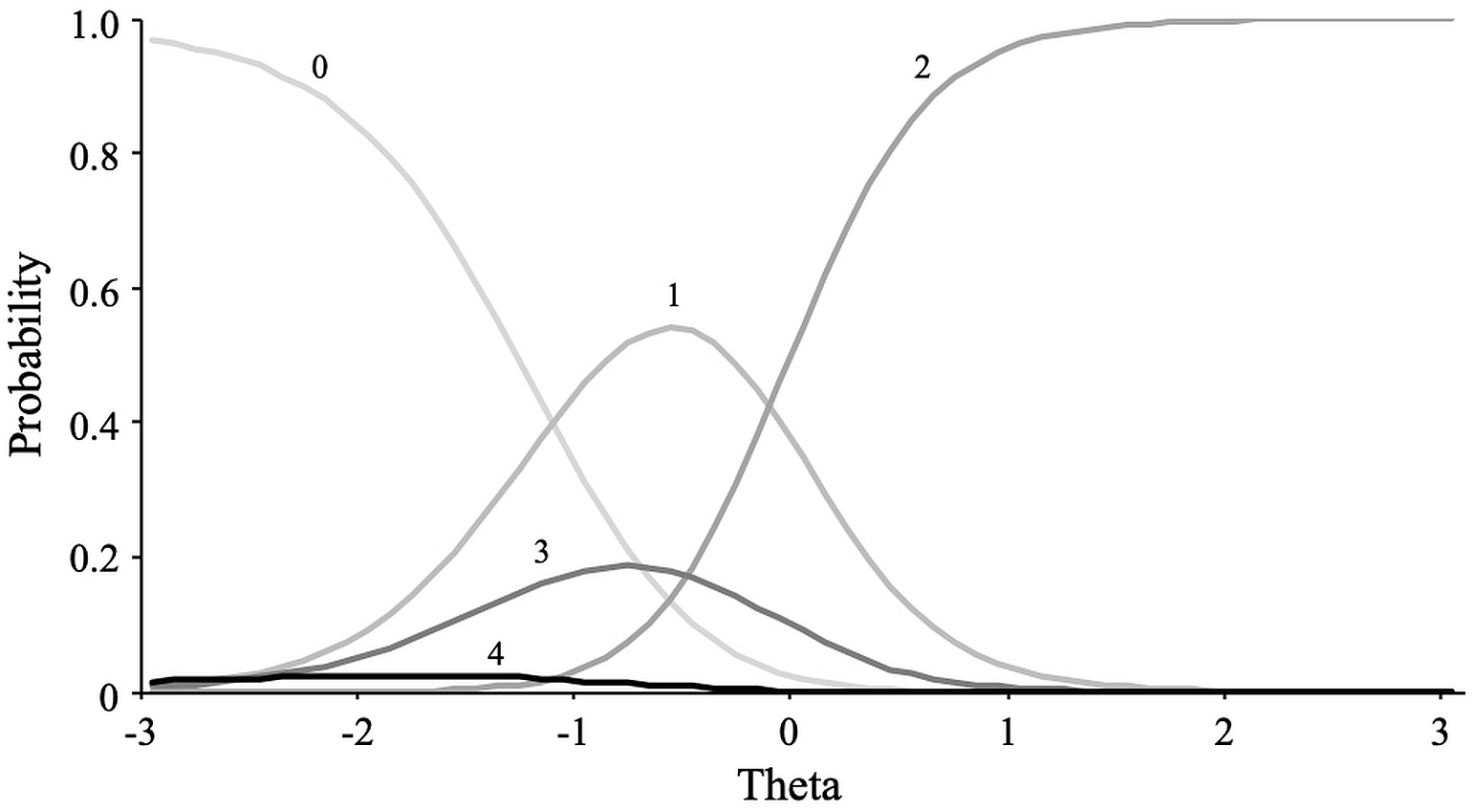
Item category curves of item “Cethonit”.

In contrast, for the whatever scale, the nominal model fitted was substantially better than the GRM model, revealing a non-constant relationship between the hypothetical trait (whateverness) and response categories. Figure 8 shows the category response curves for the item “cethonit.” Except for the first category (i.e., “Very inaccurate”), defined as the anchor category, all category response curves showed a disordered localization among the theta scores. This pattern is incompatible with what is expected of an ordinal variable.

## Discussion

In the two studies, our objective was to investigate the phenomenon described by Maul (2017) and to explore why scales with apparently meaningless items yield highly structured and consistent data, aspects traditionally associated with high validity and reliability. In the first study, we applied a set of items (mindset-gavagai scale) in which only the key term was incomprehensible and then asked the participants if they had assigned a meaning to the term “gavagai”. In the second study, we applied a scale composed of fake adjectives and compared their structure with a well-known scale consisting of understandable adjectives. In both studies, we were able to replicate Maul’s (2017) results, suggesting that the “gavagai effect” could be generalizable between samples, and while we could not establish generalizability, the effect may be extended to other instruments. The results of both studies will be discussed below.

In Study 1, we asked the participants whether they had assigned any meaning to the term “gavagai” when answering the items. This information helped us interpret some relevant aspects of the data structure observed for the complete sample. First, the apparent two-factor structure of the complete dataset (also reported by Maul (2017)) would be fictitious and promoted by a subgroup of respondents (12.7%) who claimed not to have assigned any interpretation to the term “gavagai”. As derived from the responses of some participants, this group responded to the scale in a straightline style (e.g., “I responded all yes”) or responded consistently to the item’s polarity according to understandable parts of the statement (e.g., “I tried to be consistent”). This mixture of responses unrelated to the content generates a subset of data with a large amount of artifactual variance, produced by the incoherent responses to direct and reverse-keyed items (a common anomaly in very low-quality data; Curran, 2016; Podsakoff et al., 2003). Second, the remaining identified subgroups interpreted gavagai mainly as personality or intelligence (and a large subset of participants had very diverse interpretations). For these subgroups, we obtained a clear single-factor structure. This result is not surprising because the eight items used were essentially the same question repeated with slight differences in wording. Consequently, the resulting highly structured and consistent data must be taken as evidence of both the interchangeability and interpretability of the item content and not as sufficient evidence of excellent psychometric properties.

Possibly the most relevant effect was the disparity in the observed correlation between the mindset-gavagai and mindset-intelligence scores according to the interpretation of gavagai. For the intelligence group, the association between the gavagai and intelligence scores was almost perfect (*r* = 0.94), consistent with the idea of two empirically indistinguishable scales. For the personality group, the correlation was moderately high (*r* = 0.65), mirroring general personal beliefs about the malleability of individual attributes (Dweck, 2008). However, for the null group, the correlation was quite low (*r* = 0.21), although a meaningful interpretation of this correlation is difficult, or even impossible, given the non-content-based response processes involved in this group. These results also reveal that the moderate association observed in the full sample (*r* = 0.54; similar to that reported by Maul) could be interpreted as a weighted mixture of correlations from the identified groups.

From the results of the study 1, we can conclude that (a) there is no evidence that the gavagai items lacked meaning for the respondents except in the group that did not provide an interpretation (whose data, however, showed very different properties than the data of the groups that did interpret gavagai), and (b) even in meaningless items, it is possible to detect meaningful response processes that explain the homogeneity of the response behavior throughout the items. As mentioned in the introduction, perhaps the fact that the gavagai items are mostly understandable made it easier for the respondents to interpret the only unknown term in each sentence.

In Study 2, we compared the properties of three scales to examine the differences between responses to meaningful items (extraversion adjectives; Goldberg, 1992), meaningless items (fake adjectives), and items that were meaningful but grouped without the intention of measuring any continuous latent variable (Greenleaf, 1992). For the Greenleaf scale, this set of meaningfully different items acquired neither factor structure nor internal consistency. This result is consistent with the idea that significant differences in item content lead participants to provide meaningfully different responses across items (Rhemtulla, Borsboom & Van Bork, 2017). The extraversion scale showed a poor fit to the single-factor model, which was greatly improved after introducing post hoc specifications. However, the scale composed of fake adjectives showed excellent fit to the single-factor model. Since this result is for a scale consisting of nonsense items, we could interpret it to mean that the factor analysis, like any analysis, does not distinguish the origin or meaning of the data. From a statistical point of view, the fundamental purpose of factor analysis is to describe, if possible, the variance/covariance relationships among a greater number of observed variables in terms of a smaller number of underlying latent variables (factors), regardless of where the data come from. In other words, the factor model allows us to estimate the shared variance between items. Nevertheless, it does not imply, by default, a specific interpretation of the nature of this shared variance (Markus & Borsboom, 2013). For this reason, researchers must examine and elaborate an adequate interpretation of the origin or meaning of the data and evaluate the appropriateness of a specific method for their research purposes. Additionally, the results of Study 2 support Maul’s suggestion that favorable-looking results might be regarded as a default expectation for covariance analysis on survey data. However, the results of the Greenleaf scale also suggest that there are cases in which factor analysis properly informs the absence of dimensionality in the data. The conditions under which factor analysis could lead to a “false positive” should be further investigated.

This results lead us to conclude that the interpretation of the factor as a reflection of an unobservable attribute is not correct by default. It should be subject to the null hypothesis that the factor does not represent a continuous latent variable. The same could be said of the data structure. In essence, even a perfect fit of the factor model does not imply that the structure is dimensional, so the data structure must be understood as a research hypothesis (Markus, 2008a; Markus, 2008b). For the meaningless items, we found that the three-class categorical model fit even better than the excellent-fitting single-factor model. An examination of the distribution of responses by the identified classes revealed that the participants responded to meaningless items according to the straightline and random-like response styles, in which the sum scores lose any meaning relative to any continuous latent variable. In short, the meaningless items did not measure anything except (perhaps) the preference for a specific response style. This result was supported by the disordered sequence in which the respondents used the response categories. Based on this finding, we were not able to reject the null hypothesis that meaningless items do not represent an unobservable continuum. For the extraversion scale, however, it was possible to reject the null hypothesis since the dimensional structure fit better than the categorical structure, and the empirical distribution of the response categories was consistent with the expected sequence. This result does not imply, of course, that we must accept the existence of something called extraversion that causes assertive and adventurous behaviors based only on the results of a factor analysis. It means only that a latent entity with causal power is a plausible hypothesis, among other possible explanations.

## Conclusions

In this paper, we have shown how latent variable models can be used to test hypotheses regarding the internal data structure. Of course, we are not ensuring that the categorical model is the best way to explain responses to meaningless items. However, we reported evidence for discarding the common factor model, despite its excellent fit. In this respect, it is not appropriate to attribute falsifying properties to factor analysis used alone in the absence of alternative modeling hypotheses. The problem is not that factor analysis can result in a false positive. This statistical procedure was not designed to falsify hypotheses without contrasting alternative models or to distinguish different continuous structures (for example, latent models of causal and reflective indicators; Rhemtulla, Van Bork & Borsboom, 2019). In conclusion, the potential problem is not in the method but in the use we make of the method and in our expectations of what the method is capable of doing. If we put erroneous models into the machine, it will not return correct results, even if they seem to be correct.

Throughout two studies, we have attempted to discuss some of Maul’s (2017) conclusions. We agree with several of his reflections about the need to overcome an essentially operationalist approach, to increase rigor in the use of psychometric procedures, and to understand the existence, measurability, and causal power of psychological attributes, not as truths by default but mainly as hypotheses. Additionally, in agreement with Maul, we believe that researchers’ reluctance to take a step beyond traditional psychometric validation strategies (and thus achieve the connection between psychology and psychometry proposed by Borsboom, 2006) may be indicative of a general misunderstanding of what statistical models can and cannot do. That incomprehension may lead to what computer science has summed up well in the phrase “garbage in, gospel out,” referring to uncritical and unjustified faith in the results of any statistical analysis as long as every fit is proper (Ault, 1987). A good statistical fit, in the absence of a framework of knowledge and thought that allows the meaningful interpretation of the measurement model (even if such interpretation is wrong or can be improved), is no more valid or useful for the advancement of scientific knowledge than Russell’s teapot or Sagan’s dragon in the garage. Moreover, the problem goes beyond the conceptual: fitting data to a common factor model when they do not come from a common factor model can lead to extreme biases in the estimation of parameters and the interpretation of results (Rhemtulla, Van Bork & Borsboom, 2019).

In conclusion, the method must be an instrument at the service of the researcher, and to achieve this, it is necessary to improve our understanding of the method and its limits. Otherwise, the role of the social science researcher may be reduced to producing blind ad hoc speeches to justify the results of data analysis.

##  Supplemental Information

10.7717/peerj.10209/supp-1 Supplemental Information 1 Supplementary analyses

10.7717/peerj.10209/supp-2 Supplemental Information 2 Raw data from study 1 Mindsets scale, gavagai scale, and classification of participants according to their answer to the open-ended question.

10.7717/peerj.10209/supp-3 Supplemental Information 3 Raw data from study 2 Extroversion scale, whatever scale, and Greenleaf scale.

## Additional Information and Declarations

### Data Availability

The following information was supplied regarding data availability: Raw data are available in the Supplementary Files.
