# Supplementary material for: A valid and reliable measure of nothing: disentangling the “Gavagai effect” in survey data
Source: PeerJ. 2020 Nov 17;8:e10209. doi: 10.7717/peerj.10209 (PMC7678495; doi:10.7717/peerj.10209)
Supplement: Supplemental Information 1 [file peerj-08-10209-s001.docx]

**Supplemental Material**

A valid and reliable measure of nothing: Disentangling the “Gavagai effect” in survey data

By authors

**Simulations of response vectors inattentive responders**

Here, we present a simple simulation of factor analysis fitting under conditions of a data set intentionally designed to represent four classes of respondents. The first class (n=100) emulates random respondents, by generating random responses between values 1 and 5 (assuming a 5-point Likert scale). For the second to the fourth classes, we generate random values to reproduce straightlining responding, such as disacquiescent responders (Class 2, n=33; random values between 1 and 2), middle point responders (Class 3, n=33; fixed values of 3 without variance), and acquiescent responders (Class 4, n=34; random values between 4 and 5). This sample is, for construction purposes, composed of unordered categories. Figure S1 shows the mean response histograms of 10 items of five categories from 200 simulated response vectors organized into four classes.

Figure S1. Distribution of the mean item response in four classes of inattentive respondents (simulated data).

Table S1 shows the fit we compared the fit of the factor model (continuous structure) with models that examine the presence of classes.

Table S1. Model fit of simulated data.

| **Model** | **AIC** | **BIC** | **adjBIC** |
| --- | --- | --- | --- |
| One dimensional | 5,835 | 6,000 | 5,841 |
| LCA 2 Classes | 5,599 | 5,866 | 5,609 |
| LCA 3 Classes | 5,145 | 5,548 | 5,161 |
| LCA 4 Classes | 4,892 | 5,429 | 4,913 |
| LCA 5 Classes | 4,894 | 5,567 | 4,921 |

**Note.** LCA=Latent class analysis; AIC=Akaike information criterion; BIC=Bayesian information criterion; adjBIC=Sample-size adjusted BIC.
